# Coffee and Green Tea Consumption With the Risk of COVID-19 Among the Vaccine Recipients in Japan: A Prospective Study

**DOI:** 10.2188/jea.JE20230231

**Published:** 2024-09-05

**Authors:** Zobida Islam, Shohei Yamamoto, Tetsuya Mizoue, Maki Konishi, Norio Ohmagari

**Affiliations:** 1 Department of Epidemiology and Prevention, Center for Clinical Sciences, National Center for Global Health and Medicine, Tokyo, Japan; 2 Center Hospital of the National Center for Global Health and Medicine, Tokyo, Japan

**Keywords:** coffee consumption, green tea consumption, COVID-19, vaccine, Omicron, Japanese

## Abstract

**Background:**

While coffee and green tea have been suggested to have immunoprotective effects, it remains elusive whether they can decrease the risk of coronavirus disease 2019 (COVID-19).

**Objective:**

We prospectively examined the associations of coffee and green tea consumption with the risk of COVID-19 among mRNA vaccine recipients during the epidemic of the Omicron variant.

**Methods:**

Participants were 2,110 staff (aged 18 to 76 years) of a large medical facility in Tokyo, who attended a serosurvey in June 2022, predominantly received ≥3 doses of vaccine, and were followed for COVID-19 until December 2022. Coffee and green tea consumption was ascertained via a questionnaire. COVID-19 was identified through the in-house registry. Cox proportional hazards model was used to estimate the hazard ratios (HRs) of COVID-19 across the categories of beverage consumption.

**Results:**

During 6 months of follow-up, 225 (10.6%) cases of COVID-19 were identified. Contrary to the expectation, higher consumption of coffee was associated with a significant increase in the risk of COVID-19; multivariable-adjusted HRs were 1.00 (reference), 0.92 (95% confidence interval [CI], 0.62–1.35), 1.48 (95% CI, 0.99–2.22), and 1.82 (95% CI, 1.20–2.76) for <1 cup/day, 1 cup/day, 2 cups/day, and ≥3 cups/day, respectively (*P* trend = 0.003). Green tea consumption was not significantly associated with the risk of COVID-19. The association with coffee was attenuated if serologically detected infection was added to the cases.

**Conclusion:**

In a cohort of Japanese hospital staff who received COVID-19 vaccine, higher consumption of coffee was associated with an increased risk of COVID-19 during the epidemic of the Omicron variant. There was no evidence of a significant association between green tea consumption and COVID-19 risk.

## INTRODUCTION

The global pandemic of coronavirus disease 2019 (COVID-19), caused by severe acute respiratory syndrome-related coronavirus 2 (SARS-CoV-2), has resulted in over 756 million cases and 6.84 million deaths (as of 17 February 2023).^1^ Besides infection prevention practices, such as wearing masks, hand hygiene, and social distancing, identifying modifiable risk factors for COVID-19 is of paramount importance. As regards health-related lifestyle, smoking and alcohol drinking have been suggested to increase the risk of COVID-19,^2^^–^^4^ whereas physical activity and a healthy diet have been linked to lower risk of this infection.^5^^,^^6^

Coffee and green tea have been suggested to play a role in the prevention of infectious diseases, including COVID-19. An experimental study showed that chlorogenic acid, which is found in coffee, significantly inhibits the interaction between the SARS-CoV-2 spike protein of the coronavirus and the ACE-2 receptor, the docking site for the virus on the human cell.^7^ Moreover, epigallocatechin-3-gallate (ECGC) in green tea extracts can inhibit the activity of SARS-CoV-2 Omicron variant.^8^ However, epidemiological evidence is scarce and inconsistent on the associations of coffee and green tea consumption with the risk of COVID-19. In a United Kingdom Biobank cohort among the general population, an earlier study during the epidemic of the Alpha (B.1.1.7) variant before the vaccine rollout, reported a decreased risk of COVID-19 among those who consumed higher coffee consumption (≥4 cups/day),^9^ whereas a later study using the Mendelian randomization showed that coffee consumption was associated with an increased risk of COVID-19 susceptibility and severity.^10^ In a Japanese study among staff at a medical research center during and before the Delta variant epidemic,^11^ there was a suggestion of a lower risk of infection (not statistically significant) associated with high green tea consumption (≥4 cups/day). It remains elusive, however, whether coffee and green tea consumption is associated with the risk of COVID-19 during the epidemic of the highly transmissible Omicron variant^12^ among recipients of booster vaccine, a potential modifier of the effect of risk factors which have been primarily identified for pre-Omicron infection.^13^

The objective of the present study is to examine the associations of coffee and green tea consumption with COVID-19 risk (including undiagnosed infection) among healthcare workers in Japan, where green tea consumption is high^14^ and patients with COVID-19 surged during the epidemic of the Omicron variant.^1^

## METHODS

### Study setting

The present study is a prospective study using the data from a repeat serological study which was launched during the COVID-19 pandemic (July 2020) among National Center for Global Health and Medicine (NCGM) staff to monitor the spread of COVID-19. As of March 2023, seven surveys were completed in Toyama (located in central Tokyo, approximately 2,500 staff) and three in Kohnodai areas (located in western Chiba, approximately 700 staff). In each survey, anti-SARS-CoV-2 nucleocapsid- and spike- (from the second survey onward) protein antibodies was measured using the Abbott and Roche assays and collected information on history of SARS-CoV-2 vaccination and infection, body composition, morbidity status, and behavioral factors. Self-reported vaccination status was confirmed with the record kept by the administrative department (for those who received the vaccine at NCGM), and the self-reported history of SARS-CoV-2 was validated against in-house registry maintained by the NCGM Hospital Infection Prevention and Control Unit. Written informed consent was obtained from all the participants. This study was approved by the NCGM ethics committee (approval number: NCGM-G-003598).

### Analytic cohort

**Figure 1.**
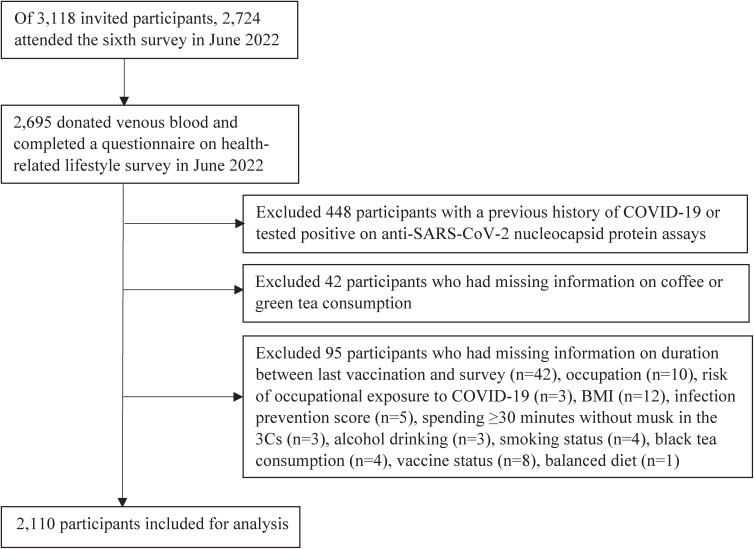
Flowchart of the study population. BMI, body mass index; COVID-19, coronavirus disease 2019; SARS-CoV-2, severe acute respiratory syndrome-related coronavirus 2.

We set a cohort of participants of the sixth survey (June 2022). Of 3,118 participants invited, 2,724 (85.6%) attended the survey (Figure 1). We excluded those with a previous history of COVID-19, who tested positive on anti-SARS-CoV-2 nucleocapsid protein assays (positive with Abbott and/or Roche), or who had missing data on coffee or green tea consumption and covariates at baseline.

### Assessment of coffee consumption and covariates

We asked the participants about the frequency of coffee and green tea consumption with six response options ranging from “don’t drink or less than 2 days per week” to “>4 cups per day” and categorized coffee consumption into four (<1 cup/day, 1 cup/day, 2 cups/day, or ≥3 cups/day) and green tea consumption into three (<1 cup/day, 1 to 2 cups/day, or ≥3 cups/day), after having taken consumption distribution among the study participants into account.

Factors considered as covariates included baseline age,^15^^,^^16^ sex,^15^^,^^16^ days after vaccination,^16^ occupations,^16^^,^^17^ risk of occupational exposure to COVID-19,^17^ infection prevention score,^18^ the frequency of spending ≥30 minutes without mask in the 3Cs (closed spaces, crowded places, and close-contact settings),^15^ the frequency of having dinner with ≥5 people for ≥1 hour,^15^ the number of household members,^18^ balanced meal consumption,^16^ smoking,^16^ alcohol drinking,^19^ the use of public transportation,^20^ body mass index (BMI),^15^^,^^16^ co-morbid conditions,^15^^,^^16^ SARS-CoV-2 spike antibody titer,^21^ and black tea consumption,^22^ which could influence the risk of COVID-19.^15^^–^^22^

We collected information on occupation, health-related lifestyles, and COVID-19-related data via a self-administered questionnaire at baseline. As regards the infection prevention scores, participants were asked about their adherence to five infection prevention practices in the past month: (1) avoiding crowded place, close-contact settings, and confined spaces; (2) social distancing; (3) wearing a mask when talking or indoors; (4) practicing good cough etiquette; and (5) washing or sanitizing hands. Each question had four response option: not at all, rarely, often, or always. Zero was assigned to “not at all” and “rarely”, 1 to “often”, and 2 to “always”. The total infection prevention scores were then calculated on a scale ranging from 0 to 10. Balanced meal consumption was assessed using the following question: How many days per week do you eat at least two meals a day comprising a staple food (eg, rice, bread, and noodles), main dish (eg, fish, meat, eggs, and soy products), and side dish (eg, vegetables, mushrooms, potatoes, and algae)? Response options were ≤1 day/week, 2–3 days/week, 4–5 days/week, or daily. As regards co-morbid conditions, participants were asked if they had any of the following chronic conditions: diabetes, hypertension, chronic obstructive pulmonary disease, heart disease, cerebrovascular disease, cancer, and other chronic diseases. Daily alcohol consumption was estimated by the frequency (ranging from never to daily) and the amount consumed per day (ranging from <0.5 to ≥4 *go*/day; go [180 mL] is used as the conventional unit to measure alcohol volume; 1 *go* Japanese sake contains approximately 23 g of ethanol, which is equivalent to 500 mL of beer, 110 mL of shochu [25% alcohol content], double [60 mL] of whisky, or 180 mL of wine).

We qualitatively measured antibody titers against the receptor-binding domain (RBD) of the SARS-CoV-2 spike protein using the AdviseDx SARS-CoV-2 IgG II assay using the Abbott ARCHITECT^®^ (immunoglobulin [Ig] G [IgG]) and the Elecsys^®^ Anti-SARS-CoV-2 S RUO (Roche). BMI was computed as weight in kilograms divided by height in meters squared.

### Identification of COVID-19

We identified COVID-19 that occurred from the six surveys (baseline) through December 31, 2022 based on the in-house registry. While most registered cases were laboratory-confirmed (polymerase chain reaction [PCR] or antigen test), some were diagnosed on clinical grounds alone without laboratory confirmation (ie, symptoms suggestive of COVID-19 following close contact with a patient with COVID-19). The registry data included the date of diagnosis, diagnostic procedure, possible route of infection (close contact person), symptoms, hospitalization, and return to work for all cases, and virus strain and cycle threshold (Ct) values for those who were diagnosed at the NCGM. We also identified the infection serologically. Specifically, we qualitatively measured antibodies against SARS-CoV-2 nucleocapsid protein using the SARS-CoV-2 IgG assay (Abbott) and Elecsys^®^ AntiSARS-CoV-2 RUO (Roche) and defined COVID-19 if the results were positive on either Abbott (≥1.0 S/C) or Roche (≥1.4 cut-off index) assays at seventh survey.

### Statistical analysis

Due to the observational nature of the current study, a formal calculation of the sample size was not applicable. As missing data was only 5.0% and the proportion of the participants with missing data did not differ across coffee consumption (eTable 1), we used a listwise deletion approach to address missing data and included only participants with complete data in analyses. Proportions and means were presented to show the baseline characteristics of the study population according to the categories of coffee consumption. Person-time was calculated from the date of receiving the health check-up to the date of COVID-19 diagnosis, the date of receiving the vaccine during follow-up, or the end of follow-up, whichever occurred first.

The Cox proportional hazards model was used to estimate the hazard ratios (HRs) of COVID-19 across the categories of coffee and green tea consumption. Model 1 was unadjusted, and model 2 was adjusted for age (years, continuous), sex (male or female), and duration between the last vaccination and baseline survey (days, continuous). Model 3 was additionally adjusted for occupation (doctors, nurses, allied healthcare professionals, administrative staff, researchers, and others), and risk of occupational exposure to COVID-19 (low, middle, or high), smoking status (never smoker, former smoker, occasional smoker, or current smoker), BMI (kg/m^2^, continuous), alcohol drinking (nondrinker, occasional drinker, <1 *go*/day, or ≥1 *go*/day), infection prevention score (continuous), the use of public transportation (no or yes), the frequency of spending ≥30 minutes without musk in the 3Cs (no, 1 to 2 times, 3 to 5 times, 6 to 9 times, or ≥10 times), the frequency of having dinner with ≥5 people for ≥1 hour (no, 1 to 2 times, 3 to 5 times, 6 to 9 times, or ≥10 times), the number of household members (continuous), balanced meal consumption (rarely, 2 to 3 days/week, 4 to 5 days/week, or almost every day), co-morbid condition (yes or no), and black tea consumption (<1 cup/day, 1 to 2 cups/day, or ≥3 cups/day). Model 4 was additionally adjusted for SARS-CoV-2 spike antibody titer (arbitrary units [AU]/mL, continuous). To examine whether the associations of coffee and green tea consumption with the risk of COVID-19 differ across age, gender, alcohol drinking, smoking, BMI, comorbidities, balanced meal diet, and baseline SARS-CoV-2 spike antibody titer, we performed stratified analyses by these variables.

To include undiagnosed infections during the follow-up in the outcome, we repeated the above analysis using logistic regression among those who attended the follow-up serological survey in December 2022 (*n* = 1,342), when new infections were identified with SARS-CoV-2 N antibody test in addition to via a COVID-19 in-house registry. We performed a sensitivity analysis where vaccination during follow-up was not considered in the person-time calculation.

Coffee and green tea consumption may influence the risk of COVID-19 through their effect on humoral response to the vaccine. We, therefore, investigated the associations of coffee and green tea consumption with SARS-CoV-2 spike antibody titer at baseline. This investigation was carried out using linear regression analysis. We also assessed the effect of multicollinearity for the multivariable model on the multivariable analyses using the Variance Influence factor (VIF), and no significant effect was observed (VIF ≤2). Statistical significance was set at *P* < 0.05 for the trend and *P* < 0.1 for interaction tests. All analyses were performed using the statistical software Stata version 18.0 (StataCorp LLC, College Station, TX, USA).

## RESULTS

As shown in Figure 1, of the 2,724 participants of the sixth survey, 2,695 donated venous blood and completed the study questionnaire. Of these, we excluded those who had a history of COVID-19 or tested positive on anti-SARS-CoV-2 nucleocapsid protein assays (*n* = 448) and those who lacked data on coffee or green tea consumption (*n* = 42) or covariates (*n* = 95), leaving 2,110 participants (aged 18 to 76 years) for the analysis.

Table 1 shows the baseline characteristics of study participants according to coffee drinking status. The proportion of those drinking <1 cup/day, 1 cup/day, 2 cups/day, and ≥3 cups/day of coffee was 50.8%, 21.6%, 15.4%, and 12.2%, respectively. More frequent coffee drinkers were older and more likely to be male, doctors, current smokers, and current alcohol drinkers; they also tended to have co-morbid conditions, consume balanced meals, use public transportation while commuting to work, and live in a larger family. They were less likely to have spent ≥30 minutes without a mask in 3Cs and consume green tea; they recorded a lower median of SARS-CoV-2 spike antibody titer (described in detail in eTable 2).

**Table 1.** Participants’ baseline characteristics according to coffee drinking status (*n* = 2,110)

	Coffee drinking status				*P-value*

	<1 cup/day	1 cup/day	2 cups/day	≥3 cups/day	
Number of participants	1,071	457	325	257	
Age, years, mean (SD)	30.6 (11.9)	37.8 (12.3)	40.2 (11.5)	39.5 (11.6)	**<0.001**
Sex, men, %	24.8	28.2	33.2	44.4	**<0.001**
BMI, kg/m^2^, mean (SD)	21.7 (3.6)	21.6 (3.2)	21.8 (3.2)	22.8 (3.5)	**<0.001**
**Occupation, %**					**<0.001**
Doctor	14.5	17.7	16.0	18.7	
Nurse	45.9	28.9	25.2	21.0	
Allied healthcare workers	16.7	16.0	12.6	16.7	
Administrative staffs	11.0	16.0	20.0	16.3	
Researchers	6.7	14.6	17.3	17.9	
Others	5.2	6.8	8.9	9.4	
**Risk of occupational exposure to SARS-CoV-2, %**					0.58
Low	57.5	55.4	59.7	58.7	
Middle	25.8	29.1	28.0	22.6	
High	16.7	15.5	12.3	18.7	
Infection prevention score,^a^ mean (SD)	8.0 (1.7)	8.3 (1.5)	8.3 (1.5)	7.9 (1.8)	0.22
Use of public transportation, yes, %	53.8	61.0	64.3	60.7	**0.001**
Frequency of spending ≥30 minutes without musk in the 3Cs, none, %	76.1	81.4	83.1	82.5	**0.002**
Frequency of having dinner with ≥5 people for ≥1 hour, none, %	83.5	85.1	88.0	87.2	**0.03**
Number of households, person, median (IQR)	2 (1–3)	2 (1–3)	3 (2–4)	3 (1–4)	**<0.001**
**Balanced meal consumption, %**					**<0.001**
Rarely	24.0	16.2	12.0	15.9	
2 to 3 days/week	25.4	21.5	21.9	26.1	
4 to 5 days/week	18.5	17.9	21.8	17.9	
Almost every day	32.1	44.4	44.3	40.1	
Co-morbid conditions, yes, %	5.6	8.8	7.4	10.9	**0.004**
**Smoking status, %**					**<0.001**
Never smoker	88.2	84.0	79.7	71.6	
Former smoker	7.6	11.2	13.2	14.4	
Occasional smoker	1.4	0.6	1.9	2.7	
Current smoker	2.8	4.2	5.2	11.3	
**Alcohol drinking status, %**					**0.004**
Non-drinker	37.2	30.4	35.4	36.2	
Occasional drinker	29.9	25.4	20.0	20.6	
<1 *go*/day	24.0	30.2	33.8	31.5	
≥1 *go*/day	8.9	14.0	10.8	11.7	
**Green tea consumption, %**					0.51
<1 cup/day	72.2	57.4	58.5	69.3	
1 to 2 cups/day	11.0	27.1	32.0	18.3	
≥3 cups/day	16.8	15.5	9.5	12.4	
**Black tea consumption, %**					0.30
<1 cup/day	88.9	86.9	85.5	92.2	
1 to 2 cups/day	7.8	11.8	13.9	5.5	
≥3 cups/day	3.3	1.3	0.6	2.3	
**Vaccine frequency, %**					0.69
1 to 2 times	3.9	4.8	4.3	5.5	
3 times	94.5	91.9	92.3	93.4	
4 or more time	1.6	3.3	3.4	1.1	
Duration between the last vaccination and baseline survey, days, median (IQR)	174 (152–186)	176 (155–186)	176 (156–183)	179 (158–184)	0.16
SARS-Cov-2 spike antibody titer, median (IQR), AU/mL	5,312 (2,837–9,877)	4,889 (2,760–9,522)	4,322 (2,239–8,441)	4,972 (2,595–9,179)	**0.01**

AU, arbitrary units; BMI, body mass index; IQR, interquartile range SARS-CoV-2, severe acute respiratory syndrome-related coronavirus 2; SD, standard deviation; SE, standard error.

^a^Infection prevention score ranged from 0 to 10. Participants who reported “always” of the five items related to infection prevention measures (avoid 3Cs (“crowded places,” “close-contact settings,” and “confined and enclosed spaces”); social distancing (2 meters; 1 meter if not possible); wear a mask when talking or when you are indoors; practice good cough etiquette; wash or sanitize hands when you return home) received two points and participants who reported “often”, received one point.

As shown in Table 2, a total of 225 participants (of whom 213 participants had completed the third or fourth doses of vaccination) tested positive for COVID-19 through in-house PCR records. A positive association, albeit statistically not significant, was found between coffee consumption and COVID-19 incident in model 1; unadjusted HRs were 1.00 (reference), 0.81 (95% confidence interval [CI], 0.56–1.17), 1.15 (95% CI, 0.80–1.66), and 1.26 (95% CI, 0.86–1.85) for <1 cup/day, 1 cup/day, 2 cups/day, and ≥3 cups/day coffee, respectively. After adjusting for age, sex, and duration of time between the last vaccination and baseline survey in model 2, these associations became stronger and statistically significant; multivariable-adjusted HRs were 1.00 (reference), 1.01 (95% CI, 0.70–1.47), 1.63 (95% CI, 1.11–2.39), and 1.76 (95% CI, 1.18–2.64) for <1 cup/day, 1 cup/day, 2 cups/day, and ≥3 cups/day coffee, respectively (*P* for trend = 0.001). After additional adjustment of all the potential covariates in model 4, the associations remain statistically significant and higher consumption of coffee was significantly associated with an increased risk of COVID-19; multivariable-adjusted HRs were 1.00 (reference), 0.92 (95% CI, 0.62–1.35), 1.48 (95% CI, 0.99–2.22), and 1.82 (95% CI, 1.20–2.76) for <1 cup/day, 1 cup/day, 2 cups/day, and ≥3 cups/day coffee, respectively (*P* for trend = 0.003). Green tea was not appreciably associated with the risk of COVID-19; multivariable-adjusted HRs were 1.00 (reference), 1.05 (95% CI, 0.73–1.52), and 0.94 (95% CI, 0.62–1.42) for consumptions of green tea <1 cup/day, 1 to 2 cups/day, and ≥3 cups/day, respectively.

**Table 2.** Hazard ratio (95% CI) of PCR-confirmed COVID-19 incidence according to the categories of coffee and green tea consumption^a^

	Number of participants	Number of Cases	Person-days	COVID-19 risk			

				Model 1	Model 2	Model 3	Model 4
**Coffee consumption**							
<1 cup/day	1,071	113	133,205	1.00 (Reference)	1.00 (Reference)	1.00 (Reference)	1.00 (Reference)
1 cup/day	457	39	55,445	0.81 (0.56–1.17)	1.01 (0.70–1.47)	0.92 (0.62–1.35)	0.92 (0.62–1.35)
2 cups/day	325	39	39,555	1.15 (0.80–1.66)	**1.63 (1.11–2.39)**	**1.50 (1.01–2.25)**	1.48 (0.99–2.22)
≥3 cups/day	257	34	30,810	1.26 (0.86–1.85)	**1.76 (1.18–2.64)**	**1.84 (1.21–2.79)**	**1.82 (1.20–2.76)**
*P for trend* ^b^				0.22	**0.001**	**0.002**	**0.003**
**Green tea consumption**							
<1 cup/day	1,404	157	173,272	1.00 (Reference)	1.00 (Reference)	1.00 (Reference)	1.00 (Reference)
1 to 2 cups/day	393	40	45,560	0.94 (0.66–1.34)	1.12 (0.79–1.60)	1.07 (0.74–1.54)	1.05 (0.73–1.52)
≥3 cups/day	313	28	40,183	0.78 (0.52–1.17)	0.88 (0.58–1.31)	0.94 (0.62–1.42)	0.94 (0.62–1.42)
*P for trend* ^b^				0.25	0.72	0.85	0.85

CI, confidence interval; COVID-19, coronavirus 2019; PCR, polymerase chain reaction.

Model 1 was unadjusted. Model 2 was adjusted for age (year, continuous), sex (male or female), and duration between the last vaccination and baseline survey (days, continuous). Model 3 was additionally adjusted for occupation (doctors, nurses, allied healthcare professionals, administrative staff, researchers, and others), risk of occupational exposure to COVID-19 (low, middle, or high), cigarette smoking (never smoker, former smoker, occasional smoker, or current smoker), body mass index (kg/m^2^, continuous), alcohol drinking (nondrinker, occasional drinker, <1 *go*/day, or ≥1 *go*/day), infection prevention score (continuous), use of public transportation (no or yes), frequency of spending ≥30 minutes without musk in the 3Cs (no, 1 to 2 times, 3 to 5 times, 6 to 9 times, or ≥10 times), frequency of having dinner with ≥5 people for ≥1 hour (no, 1 to 2 times, 3 to 5 times, 6 to 9 times, or ≥10 times), black tea consumption (<1 cup/day, 1 to 2 cups/day, or ≥3 cups/day), number of household (continuous), balanced meal consumption (rarely, 2 to 3 days/week, 4 to 5 days/week, or almost every day), and co-morbid conditions (yes or no). Model 4 was additionally adjusted for severe acute respiratory syndrome-related coronavirus-2 spike antibody titer (AU/mL, continuous).

^a^For coffee consumption model was additionally adjusted for green tea consumption (<1 cup/day, 1 to 2 cups/day, or ≥3 cups/day), and for green tea consumption model was additionally adjusted for coffee consumption (<1 cup/day, 1 cup/day, 2 cups/day, or ≥3 cups/day).

^b^Based on Cox regression analysis, assigning ordinal numbers to the coffee and green tea consumption status.

A higher risk of COVID-19 infection associated with higher coffee consumption was observed in all subgroups stratified by age (<40 years or ≥40 years), gender, BMI (<23 kg/m^2^ or ≥23 kg/m^2^), alcohol drinking (non-drinkers or drinkers), balanced diet (almost every day or <6 days/week), baseline SARS-CoV-2 spike antibody titer (higher or lower median), as well as among non-smokers or individuals without comorbidities (Table 3), with no indication of significant interaction (*P* for interaction > 0.2) with age, gender, BMI, alcohol drinking, balanced diet, and baseline SARS-CoV-2 spike antibody titer. Green tea consumption was not associated with the risk of COVID-19 infection in any subgroup (Table 4).

**Table 3.** Multivariable adjusted hazard ratio (95% CI) of PCR-confirmed COVID-19 incidence for coffee consumption across subgroups

	Coffee consumption				*P* for trend	*P* for interaction

	<1 cup/day	1 cup/day	2 cups/day	≥3 cups/day		
**Gender**						
**Male (*n* = 617)**	266	129	108	114		
Number of cases	25	11	13	13		
Multivariable-adjusted model	1.00 (Reference)	0.83 (0.38–1.83)	1.47 (0.70–3.09)	1.50 (0.73–3.09)	0.18	
**Female (*n* = 1,493)**	805	328	217	143		
Number of cases	88	28	26	21		
Multivariable-adjusted model	1.00 (Reference)	0.93 (0.59–1.46)	1.52 (0.93–2.49)	**2.07 (1.24–3.45)**	**0.005**	0.30
**Age**						
**<40 years (*n* = 1,138)**	752	196	100	90		
Number of cases	96	24	12	19		
Multivariable-adjusted model	1.00 (Reference)	0.91 (0.57–1.45)	0.96 (0.51–1.79)	**1.71 (1.01–2.89)**	0.15	
**≥40 years (*n* = 972)**	319	261	225	167		
Number of cases	17	15	27	15		
Multivariable-adjusted model	1.00 (Reference)	1.00 (0.48–2.04)	**2.22 (1.16–4.23)**	1.75 (0.83–3.71)	**0.02**	0.34
**Alcohol drinking status**						
**Non-drinker (*n* = 746)**	399	139	115	93		
Number of cases	36	8	10	10		
Multivariable-adjusted model	1.00 (Reference)	0.59 (0.26–1.37)	1.08 (0.48–2.42)	2.10 (0.95–4.66)	0.15	
**Drinker (*n* = 1,364)**	672	318	210	164		
Number of cases	77	31	29	24		
Multivariable-adjusted model	1.00 (Reference)	1.02 (0.65–1.60)	**1.75 (1.08–2.81)**	**1.90 (1.15–3.15)**	**0.004**	0.73
**Smoking** ^a^						
**Non-smokers (*n* = 1,984)**	1,026	435	302	221		
Number of cases	110	39	38	32		
Multivariable-adjusted model	1.00 (Reference)	0.92 (0.63–1.36)	1.47 (0.98–2.21)	**1.82 (1.19–2.78)**	**0.003**	
**Comorbidity** ^a^						
**No (*n* = 1,958)**	1,011	417	301	229		
Number of cases	109	38	37	32		
Multivariable-adjusted model	1.00 (Reference)	0.92 (0.62–1.36)	1.48 (0.98–2.23)	**1.82 (1.19–2.78)**	**0.003**	
**Balanced meal consumption**						
**Almost every day (*n* = 794)**	344	203	144	103		
Number of cases	42	21	19	19		
Multivariable-adjusted model	1.00 (Reference)	1.00 (0.57–1.76)	1.42 (0.78–2.58)	**2.21 (1.22–3.98)**	**0.01**	
**<6 days/week (*n* = 1,316)**	727	254	181	154		
Number of cases	71	18	20	15		
Multivariable-adjusted model	1.00 (Reference)	0.86 (0.50–1.46)	1.69 (0.96–2.96)	1.63 (0.89–2.98)	0.05	0.43
**BMI** ^b^						
**Normal weight (*n* = 1,512)**	797	334	236	145		
Number of cases	87	32	27	22		
Multivariable-adjusted model	1.00 (Reference)	0.97 (0.63–1.50)	1.35 (0.84–2.16)	**1.93 (1.17–3.19)**	**0.01**	
**Overweight (*n* = 598)**	274	123	89	112		
Number of cases	26	7	12	12		
Multivariable-adjusted model	1.00 (Reference)	0.82 (0.34–1.99)	2.03 (0.91–4.55)	1.74 (0.80–3.38)	0.10	0.67
**Baseline SARS-Cov-2 spike antibody titer** ^c^						
**Lower median (*n* = 1,055)**	511	233	181	130		
Number of cases	58	24	26	20		
Multivariable-adjusted model	1.00 (Reference)	1.05 (0.63–1.75)	**1.81 (1.06–3.07)**	**1.97 (1.13–3.43)**	**0.005**	
**Higher median (*n* = 1,055)**	560	224	144	127		
Number of cases	55	15	13	14		
Multivariable-adjusted model	1.00 (Reference)	0.74 (0.41–1.35)	1.16 (0.61–2.21)	1.61 (0.85–3.04)	0.20	0.72

AU, arbitrary units; BMI, body mass index; CI, confidence interval; COVID-19, coronavirus disease 2019; PCR, polymerase chain reaction; SARS-CoV-2, severe acute respiratory syndrome-related coronavirus 2.

For age, gender and drinking status model was mutually adjusted for age (year, continuous), sex (male or female), duration between the last vaccination and baseline survey (days, continuous), occupation (doctors, nurses, allied healthcare professionals, administrative staff, researchers, and others), risk of occupational exposure to COVID-19 (low, middle, or high), cigarette smoking (never smoker, former smoker, occasional smoker, or current smoker), body mass index (kg/m^2^, continuous), alcohol drinking (nondrinker, occasional drinker, <1 *go*/day, or ≥1 *go*/day), infection prevention score (continuous), use of public transportation (no or yes), frequency of spending ≥30 minutes without musk in the 3Cs (no, 1 to 2 times, 3 to 5 times, 6 to 9 times, or ≥10 times), frequency of having dinner with ≥5 people for ≥1 hour (no, 1 to 2 times, 3 to 5 times, 6 to 9 times, or ≥10 times), black tea consumption (<1 cup/day, 1 to 2 cups/day, or ≥3 cups/day), number of household (continuous), balanced meal consumption (rarely, 2 to 3 days/week, 4 to 5 days/week, or almost every day), green tea consumption (<1 cup/day, 1 to 2 cups/day, or ≥3 cups/day), co-morbid conditions (yes or no), and SARS-CoV-2 spike antibody titer (AU/mL, continuous).

^a^Because of the low numbers of smokers [1.5% occasionally smoked somedays, 4.5% smoked every day] and participants with comorbidities (7.2%), we conducted the analysis only among non-smokers and among those with no comorbidities.

^b^Body mass index was categorized as normal weight (<23 kg/m^2^) or overweight (≥23 kg/m^2^) according to the World Health Organization classification for Asians.

^c^The cut-off for the lower or higher median of baseline SARS-Cov-2 spike antibody titer was ≤5,003 AU/mL or >5,003 AU/mL.

**Table 4.** Multivariable adjusted hazard ratio (95% CI) of PCR-confirmed COVID-19 incidence for green tea consumption across subgroups

	Green tea consumption			*P* for trend	*P* for interaction

	<1 cup/day	1 to 2 cups/day	≥3 cups/day		
**Gender**					
**Male (*n* = 617)**	389	132	96		
Number of cases	46	9	7		
Multivariable-adjusted model	1.00 (Reference)	0.58 (0.26–1.28)	0.80 (0.34–1.85)	0.32	
**Female (*n* = 1,493)**	1,015	261	217		
Number of cases	111	31	21		
Multivariable-adjusted model	1.00 (Reference)	1.33 (0.87–2.04)	1.05 (0.65–1.69)	0.60	0.15
**Age**					
**<40 years (*n* = 1,138)**	834	158	146		
Number of cases	112	22	17		
Multivariable-adjusted model	1.00 (Reference)	1.05 (0.65–1.70)	0.83 (0.49–1.41)	0.55	
**≥40 years (*n* = 972)**	570	235	167		
Number of cases	45	18	11		
Multivariable-adjusted model	1.00 (Reference)	0.90 (0.50–1.62)	0.97 (0.48–1.96)	0.84	0.99
**Alcohol drinking status**					
**Non-drinker (*n* = 746)**	486	130	130		
Number of cases	42	11	11		
Multivariable-adjusted model	1.00 (Reference)	1.28 (0.61–2.70)	1.14 (0.58–2.27)	0.60	
**Drinker (*n* = 1,364)**	918	263	183		
Number of cases	115	29	17		
Multivariable-adjusted model	1.00 (Reference)	1.14 (0.74–1.76)	0.80 (0.48–1.36)	0.63	0.78
**Smoking status** ^a^					
**Non-smokers (*n* = 1,984)**	1,331	365	288		
Number of cases	151	40	28		
Multivariable-adjusted model	1.00 (Reference)	1.12 (0.77–1.62)	1.02 (0.67–1.54)	0.77	
**Comorbidity** ^a^					
**No (*n* = 1,958)**	1,322	355	281		
Number of cases	151	39	26		
Multivariable-adjusted model	1.00 (Reference)	1.09 (0.75–1.58)	0.92 (0.60–1.41)	0.86	
**Balanced meal consumption**					
**Almost every day (*n* = 794)**	492	163	139		
Number of cases	69	19	13		
Multivariable-adjusted model	1.00 (Reference)	0.88 (0.50–1.51)	0.89 (0.48–1.65)	0.62	
**<6 days/week (*n* = 1,316)**	912	230	174		
Number of cases	88	21	15		
Multivariable-adjusted model	1.00 (Reference)	1.27 (0.77–2.09)	1.06 (0.60–1.84)	0.62	0.45
**BMI** ^b^					
**Normal weight (*n* = 1,512)**	1,040	272	200		
Number of cases	119	29	20		
Multivariable-adjusted model	1.00 (Reference)	1.10 (0.71–1.69)	0.94 (0.58–1.54)	0.96	
**Overweight (*n* = 598)**	364	121	113		
Number of cases	38	11	8		
Multivariable-adjusted model	1.00 (Reference)	1.06 (0.50–2.24)	0.97 (0.43–2.16)	0.99	0.96
**Baseline SARS-Cov-2 antibody titer** ^c^					
**Lower median (*n* = 1,055)**	707	194	154		
Number of cases	90	21	17		
Multivariable-adjusted model	1.00 (Reference)	0.94 (0.56–1.60)	1.10 (0.64–1.89)	0.82	
**Higher median (*n* = 1,055)**	697	199	159		
Number of cases	67	19	11		
Multivariable-adjusted model	1.00 (Reference)	1.25 (0.73–2.14)	0.74 (0.39–1.42)	0.60	0.59

AU, arbitrary units; BMI, body mass index; CI, confidence interval; COVID-19, coronavirus disease 2019; PCR, polymerase chain reaction; SARS-CoV-2, severe acute respiratory syndrome-related coronavirus 2.

For age, agender and drinking status model was mutually adjusted for age (year, continuous), sex (male or female), duration between the last vaccination and baseline survey (days, continuous), occupation (doctors, nurses, allied healthcare professionals, administrative staffs, researchers, and others), risk of occupational exposure to COVID-19 (low, middle, or high), cigarette smoking (never smoker, former smoker, occasional smoker, or current smoker), body mass index (kg/m^2^, continuous), alcohol drinking (nondrinker, occasional drinker, <1 *go*/day, or ≥1 *go*/day), infection prevention score (continuous), use of public transportation (no or yes), frequency of spending ≥30 minutes without musk in the 3Cs (no, 1 to 2 times, 3 to 5 times, 6 to 9 times, or ≥10 times), frequency of having dinner with ≥5 people for ≥1 hour (no, 1 to 2 times, 3 to 5 times, 6 to 9 times, or ≥10 times), black tea consumption (<1 cup/day, 1 to 2 cups/day, or ≥3 cups/day), coffee consumption (<1 cup/day, 1 cup/day, 2 cups/day, or ≥3 cups/day), number of household (continuous), balanced meal consumption (rarely, 2 to 3 days/week, 4 to 5 days/week, or almost every day), co-morbid conditions (yes or no), and SARS-CoV-2 spike antibody titer (AU/mL, continuous).

^a^Because of the low numbers of smokers [1.5% occasionally smoked somedays, 4.5% smoked every day] and participants with comorbidities (7.2%), we conducted the analysis only among non-smokers and among those with no comorbidities.

^b^Body mass index was categorized as normal weight (<23 kg/m^2^) or overweight (≥23 kg/m^2^) according to the World Health Organization classification for Asians.

^c^The cut-off for the lower or higher median of baseline SARS-Cov-2 spike antibody titer was ≤5,003 AU/mL or >5,003 AU/mL.

In the analyses including both diagnosed and undiagnosed (detected serologically only) infection as the cases, the association for coffee was somewhat attenuated (model 3); multivariable-adjusted odds ratios were 1.00 (reference), 0.98 (95% CI, 0.69–1.38), 1.25 (95% CI, 0.84–1.87), and 1.44 (95% CI, 0.95–2.19) for <1 cup/day, 1 cup/day, 2 cups/day, and ≥3 cups/day coffee, respectively (*P* for trend = 0.08). For higher consumption of green tea (≥3 cups/day), the odds ratio of COVID-19 was decreased by >20%, albeit statistically not significant (eTable 3).

Results for coffee and green tea consumption were materially unchanged after changing the definition for censoring (including those subjects who received the COVID-19 vaccine during the follow-up) (eTable 4) and after adjusting for wearing a mask when talking or indoors HRs 1.80; 95% CI, 1.19–2.73) (data not shown in table).

## DISCUSSION

In the current study among the staff of a large referral hospital in Japan who received the mRNA vaccine, higher consumption of coffee was associated with an increased, rather than decreased, risk of COVID-19. There was no evidence of a significant association between green tea consumption and the risk of COVID-19. To the best of our knowledge, this is the first study that examined the associations of coffee and green tea consumption with COVID-19 risk after the third dose of the COVID-19 vaccination.

The present positive association between coffee consumption and the risk of COVID-19 agrees with that of a Mendelian randomization study among the United Kingdom Biobank cohort, reporting a causal relationship between coffee intake and an increased susceptibility to COVID-19.^10^ Our results are in contrast with another United Kingdom Biobank study that showed a lower risk of COVID-19 among coffee drinkers.^9^ This disparity in results between the present study and the earlier report from the United Kingdom Biobank may be attributed, at least in part, to the difference in the timing of infection relative to the vaccination and the nature of the variant. The United Kingdom study^9^ was conducted during the epidemic of the Alpha (B.1.1.7) variant before the vaccine rollout, while the current study was conducted during the epidemic of the Omicron variant after receiving the third vaccine dose.

In our explanatory analysis, higher coffee consumption was associated with lower SARS-Cov-2 spike antibody titers at baseline among the vaccine recipients, suggesting a detrimental role of coffee in the immune response to the vaccine. The adjustment of baseline antibody titer (model 4), however, did not materially alter the association between coffee drinking and COVID-19 risk, denying the possibility of the suppression of vaccine-induced immune response as an explanation for the observed association. Contrary to animal experimental data indicating anti-inflammatory effect of coffee,^23^ data in humans showed that consuming more than 200 mL of coffee per day was associated with higher levels of proinflammatory markers such as C-reactive protein, IL-6, and tumor necrosis factor α,^24^ which may suppress the immune response against viral infection.^25^^,^^26^ Further studies are required to confirm the present positive association between coffee drinking and COVID-19 risk and explore the underlying mechanism behind the association.

Despite the evidence of the vitro study indicating a protective role of green tea extract against SARS-CoV-2 Omicron variant,^8^ we found no association between green tea consumption and the risk of diagnosed COVID-19. In the analysis including both diagnosed and undiagnosed (detected serologically only) infection as the outcome, the odds of infection associated with ≥3 cups/day of green tea consumption was decreased by 24%, without statistical significance. In our previous report among those before and after the 2nd dose of COVID-19 vaccines during and prior to the Delta variant epidemic, there was a suggestive inverse association (not statistically significant).^11^ The present study was conducted during the epidemic of highly transmissive Omicron variant among those who completed the third dose of vaccines. Besides the statistical power issue, the lack of a significant association in these epidemiological studies may reflect the difference in the exposure level of ECGC, a potential inhibitor of COVID-19.^27^^,^^28^ Specifically, the concentrations of ECGC in green tea in daily life setting is much lower than those in experimental studies using green tea extracts.^29^ Additionally, ECGC is unstable and poorly absorbed when consumed orally from green tea.^30^ The present study did not provide evidence supporting a large impact of green tea consumption in the prevention of COVID-19.

The strengths of the present study included its prospective design, a cohort of well-characterized population, in-house registry for the identification of COVID-19 cases, and adjustment of a wide range of potential covariates. Besides, the present study also has some limitations that warrant mention. First, coffee and green tea consumption was self-reported and thus subject to misclassification. Second, the bioactive compounds in coffee and green tea can vary depending on the preparation method,^31^^,^^32^ but our study did not collect any information regarding the specific preparation methods used. Third, although we adjusted for a wide range of potential confounders, we cannot rule out the possibility that the observed associations are due to unmeasured and residual confounding. For example, our study lacked information on the type of accommodation people were living in (ie, house, apartment/other), which can affect infection transmission. Fourth, the present study may be underpowered to detect a modest association (in the case of green tea) with statistical significance. Finally, we examined the effect of usual coffee consumption on the risk of Omicron BA.5 infection, which was epidemic in Japan during July to September, and our study participants were apparently healthy and working in a single medical facility. Caution should be exercised when applying these findings to the other variants and populations with different backgrounds.

In conclusion, the present study suggests that higher consumption of coffee (3 cups/day or more) is associated with an increased risk of COVID-19 among 3-dose vaccine recipients during the epidemic of Omicron BA.5. There was no evidence of a significant association between green tea consumption and the risk of COVID-19.

## SUPPLEMENTARY MATERIAL

eTable 1. Percentages of subjects with missing data according to coffee-drinking status

eTable 2. Mean (95% CI) of SARS-Cov-2 spike antibody titer according to the category of coffee and green tea consumption^a^ at baseline

eTable 3. Odds ratio (95% CI) of COVID-19 according to the category of coffee and green tea consumption among those who attended the follow-up serological survey in December 2022^a^ (n = 1,342)

eTable 4. Hazard ratio (95% CI) of PCR confirmed COVID-19 incidence (including those subjects who received corona vaccine during the follow-up) according to the categories of coffee and green tea consumption^a^
