# Erratum: “Coffee and Green Tea Consumption With the Risk of COVID-19 Among the Vaccine Recipients in Japan: A Prospective Study”

**DOI:** 10.2188/jea.JE20250309

**Published:** 2025-12-05

**Authors:** Zobida Islam, Shohei Yamamoto, Tetsuya Mizoue, Maki Konishi, Norio Ohmagari

**Affiliations:** 1 Department of Epidemiology and Prevention, Center for Clinical Sciences, National Center for Global Health and Medicine, Tokyo, Japan; 2 Center Hospital of the National Center for Global Health and Medicine, Tokyo, Japan

In the original publication of this article,^1^ an error was identified in the **Methods** section under *“Identification of COVID-19”*, specifically in the final sentence of this subsection. The error was due to a typographical mistake in reporting the threshold values for the Abbott and Roche assays. Table 1 shows the erratum of the text. The authors sincerely apologize for this oversight.

**Table 1.** Erratum

**Before correction**	**After correction**
**Identification of COVID-19** We identified COVID-19 that occurred from the six surveys (baseline) through December 31, 2022 based on the in-house registry. While most registered cases were laboratory-confirmed (polymerase chain reaction [PCR] or antigen test), some were diagnosed on clinical grounds alone without laboratory confirmation (ie, symptoms suggestive of COVID-19 following close contact with a patient with COVID-19). The registry data included the date of diagnosis, diagnostic procedure, possible route of infection (close contact person), symptoms, hospitalization, and return to work for all cases, and virus strain and cycle threshold (Ct) values for those who were diagnosed at the NCGM. We also identified the infection serologically. Specifically, we qualitatively measured antibodies against SARS-CoV-2 nucleocapsid protein using the SARS-CoV-2 IgG assay (Abbott) and Elecsys^®^ AntiSARS-CoV-2 RUO (Roche) and defined COVID-19 if the results were positive on either Abbott (**≥1.0 **S/C) or Roche (**≥1.4 **cut-off index) assays at seventh survey.	**Identification of COVID-19** We identified COVID-19 that occurred from the six surveys (baseline) through December 31, 2022 based on the in-house registry. While most registered cases were laboratory-confirmed (polymerase chain reaction [PCR] or antigen test), some were diagnosed on clinical grounds alone without laboratory confirmation (ie, symptoms suggestive of COVID-19 following close contact with a patient with COVID-19). The registry data included the date of diagnosis, diagnostic procedure, possible route of infection (close contact person), symptoms, hospitalization, and return to work for all cases, and virus strain and cycle threshold (Ct) values for those who were diagnosed at the NCGM. We also identified the infection serologically. Specifically, we qualitatively measured antibodies against SARS-CoV-2 nucleocapsid protein using the SARS-CoV-2 IgG assay (Abbott) and Elecsys^®^ AntiSARS-CoV-2 RUO (Roche) and defined COVID-19 if the results were positive on either Abbott (**≥1.4** S/C) or Roche (**≥1.0** cut-off index) assays at seventh survey.
