# Supplementary material for: Coffee and Green Tea Consumption With the Risk of COVID-19 Among the Vaccine Recipients in Japan: A Prospective Study
Source: J Epidemiol. 2024 Sep 5;34(9):444–52. doi: 10.2188/jea.JE20230231 (PMC11330706; doi:10.2188/jea.JE20230231)
Supplement: Supplementary file 1 [file je-34-444-s001.pdf]

eTable 1. Percentages of subjects with missing data according to coffee-drinking status

	**Coffee drinking status**			
	<1 cup/day	1 cup/day	2 cups/day	≥3 cups/day
Total (n=2,645)	1,357	570	399	319
Duration of vaccine, %	1.8	2.5	0.3	0.9
Occupation, %	0.5	0.2	0	0.6
Risk of occupational exposure to SARS-CoV-2, %	0.07	0	0.5	0
Body mass index, %	0.4	0.4	0.5	0.6
Infection prevention behaviors, %	0.1	0.2	0.5	0
Spending ≥30 minutes without musk in the 3Cs, %	0.2	0	0	0
Alcohol drinking, %	0.07	0	0.3	0.3
Smoking status, %	0.1	0.2	0.3	0
Black tea consumption, %	0.07	0	0.8	0
Vaccine status, %	0.3	0	0.8	0.3
Balanced diet, %	0.07	0	0	0

SARS-CoV-2, severe acute respiratory syndrome-related coronavirus 2.

eTable 2. Mean (95% CI) of SARS-Cov-2 spike antibody titer according to the category of coffee and green tea consumption[a] at baseline

	SARS-Cov-2 spike antibody titer					
	Geometric means			Ratio of means		
	**Model 1**	**Model 2**	**Model 3**	**Model 1**	**Model 2**	**Model 3**
**Coffee Consumption**						
<1 cup/day	5,294 (4,988–5,620)	5,237 (4,963–5,525)	5,175 (4,902–5,464)	1.00 (Reference)	1.00 (Reference)	1.00 (Reference)
1 cup/day	5,113 (4,667–5,602)	5,271 (4,865–5,710)	5,327 (4,915–5,774)	0.97 (0.87–1.08)	1.01 (0.91–1.11)	1.03 (0.93–1.14)
2 cups/day	4,514 (4,050–5,030)	4,501 (4,090–4,953)	4,540 (4,121–5,003)	**0.85 (0.75–0.96)**	**0.86 (0.77–0.96)**	**0.87 (0.78–0.98)**
≥3 cups/day	4,656 (4,122–5,259)	4,635 (4,162–5,161)	4,723 (4,237–5,265)	0.88 (0.77–1.00)	**0.88 (0.78–0.99)**	0.91 (0.81–1.03)
P for trend[b]				**0.01**	**0.01**	**0.03**
**Green tea consumption**						
<1 cup/day	5,026 (4,770–5,295)	5,081 (4,854–5,319)	5,114 (4,885–5,353)	1.00 (Reference)	1.00 (Reference)	1.00 (Reference)
1-2 cups/day	5,120 (4,639–5,651)	5,045 (4,626–5,503)	5,030 (4,602–5,497)	1.02 (0.91–1.13)	0.99 (0.90–1.10)	0.99 (0.89–1.09)
≥3 cups/day	5,053 (4,524–5,644)	4,914 (4,451–5,397)	4,782 (4,340–5,269)	1.01 (0.89–1.14)	0.96 (0.87–1.07)	0.93 (0.84–1.04)
P for trend[b]				0.85	0.53	0.26

CI, confidence interval; COVID-19, coronavirus disease 2019; SARS-CoV-2, severe acute respiratory syndrome-related coronavirus 2.

Model 1 was unadjusted. Model 2 was adjusted for age (year, continuous), sex (male or female), and duration between the last vaccination and baseline survey (days, continuous). Model 3 was additionally adjusted for occupation (doctors, nurses, allied healthcare professionals, administrative staff, researchers, and others), risk of occupational exposure to COVID-19 (low, middle, or high), cigarette smoking (never smoker, former smoker, occasional smoker, or current smoker), body mass index (kg/m^2, continuous), alcohol drinking (nondrinker, occasional drinker, <1 go/day, or ≥1 go/day), infection prevention score (continuous), use of public transportation (no or yes), frequency of spending ≥30 minutes without musk in the 3Cs (no, 1 to 2 times, 3 to 5 times, 6 to 9 times, or ≥10 times), frequency of having dinner with ≥5 people for ≥1 hour (no, 1 to 2 times, 3 to 5 times, 6 to 9 times, or ≥10 times), number of households (continuous), balanced meal consumption (rarely, 2 to 3 days/week, 4 to 5 days/week, or almost every day), co-morbid conditions (yes or no), and black tea consumption (<1 cup/day, 1 to 2 cups/day, or ≥3 cups/day).

[a]For coffee consumption model was additionally adjusted for green tea consumption (<1 cup/day, 1 to 2 cups/day, or ≥3 cups/day), and for green tea consumption model was additionally adjusted for coffee consumption (<1 cup/day, 1 cup/day, 2 cups/day, or ≥3 cups/day).

[b]Based on linear regression analysis, assigning ordinal numbers to the coffee and green tea consumption status.

eTable 3. Odds ratio (95% CI) of COVID-19 according to the category of coffee and green tea consumption among those who attended the follow-up serological survey in December 2022[a] (n=1,342)

	Number of participants	Number of cases	Positive antibody test (either Abbott or Roche)			
			Model 1	Model 2	Model 3	Model 4
**Coffee Consumption**						
<1 cup/day	648	173	1.00 (Reference)	1.00 (Reference)	1.00 (Reference)	1.00 (Reference)
1 cup/day	308	73	0.85 (0.62–1.17)	1.11 (0.80–1.55)	0.98 (0.69–1.38)	0.97 (0.69–1.37)
2 cups/day	211	55	0.97 (0.68–1.38)	1.40 (0.95–2.05)	1.25 (0.84–1.87)	1.19 (0.80–1.78)
≥3 cups/day	175	50	1.10 (0.76–1.59)	**1.52 (1.02–2.27)**	1.44 (0.95–2.19)	1.39 (0.91–2.11)
P for trend[b]			0.78	**0.02**	0.08	0.13
**Green tea consumption**						
<1 cup/day	864	240	1.00 (Reference)	1.00 (Reference)	1.00 (Reference)	1.00 (Reference)
1 to 2 cups/day	273	69	0.88 (0.64–1.20)	1.04 (0.76–1.45)	1.06 (0.76–1.50)	1.06 (0.75–1.49)
≥3 cups/day	205	42	0.67 (0.46–0.97)	0.74 (0.51–1.08)	0.79 (0.53–1.16)	0.78 (0.53–1.15)
P for trend[b]			0.03	0.20	0.35	0.31

CI, confidence interval; COVID-19, coronavirus disease 2019.

Model 1 was unadjusted. Model 2 was adjusted for age (year, continuous), sex (male or female), and duration between the last vaccination and baseline survey (days, continuous). Model 3 was additionally adjusted for occupation (doctors, nurses, allied healthcare professionals, administrative staff, researchers, and others), risk of occupational exposure to COVID-19 (low, middle, or high), cigarette smoking (never smoker, former smoker, occasional smoker, or current smoker), body mass index (kg/m^2, continuous), alcohol drinking (nondrinker, occasional drinker, <1 *go*/day, or ≥1 *go*/day), infection prevention score (continuous), use of public transportation (no or yes), frequency of spending ≥30 minutes without musk in the 3Cs (no, 1 to 2 times, 3 to 5 times, 6 to 9 times, or ≥10 times), frequency of having dinner with ≥5 people for ≥1 hour (no, 1 to 2 times, 3 to 5 times, 6 to 9 times, or ≥10 times), number of households (continuous), balanced meal consumption (rarely, 2 to 3 days/week, 4 to 5 days/week, or almost every day), co-morbid conditions (yes or no), and black tea consumption (<1 cup/day, 1 to 2 cups/day, or ≥3 cups/day). Model 4 was additionally adjusted for SARS-CoV-2 spike antibody titer (AU/ml, continuous).

[a]For coffee consumption model was additionally adjusted for green tea consumption (<1 cup/day, 1 to 2 cups/day, or ≥3 cups/day), and for green tea consumption model was additionally adjusted for coffee consumption (<1 cup/day, 1 cup/day, 2 cups/day, or ≥3 cups/day).

[b]Based on linear regression analysis, assigning ordinal numbers to the coffee and green tea consumption status.

eTable 4. Hazard ratio (95% CI) of PCR confirmed COVID-19 incidence (including those subjects who received corona vaccine during the follow-up) according to the categories of coffee and green tea consumption[a]

	Number of participants	Number of cases	Person-days	COVID-19 risk			
				**Model 1**	**Model 2**	**Model3**	**Model 4**
**Coffee Consumption**							
<1 cup/day	1,071	113	171,241	1.00 (Reference)	1.00 (Reference)	1.00 (Reference)	1.00 (Reference)
1 cup/day	457	39	74,160	0.79 (0.55–1.14)	1.01 (0.70–1.47)	0.91 (0.62–1.34)	0.91 (0.62–1.35)
2 cups/day	325	39	51,219	1.15 (0.80–1.65)	**1.63 (1.11–2.39)**	**1.49 (1.00–2.23)**	1.48 (0.99–2.20)
≥3 cups/day	257	34	40,468	1.26 (0.86–1.85)	**1.76 (1.18–2.64)**	**1.82 (1.20–2.76)**	**1.81 (1.19–2.73)**
P for trend[b]				0.20	**0.001**	**0.002**	**0.003**
**Green tea consumption**							
<1 cup/day	1,404	157	224,187	1.00 (Reference)	1.00 (Reference)	1.00 (Reference)	1.00 (Reference)
1 to 2 cups/day	393	40	62,386	0.92 (0.65–1.30)	1.10 (0.77–1.56)	1.07 (0.74–1.54	1.07 (0.74–1.54)
≥3 cups/day	313	28	50,515	0.80 (0.53–1.19)	0.89 (0.60–1.34)	0.94 (0.62–1.41)	0.94 (0.62–1.41)
P for trend[b]				0.25	0.76	0.89	0.88

CI, confidence interval; COVID-19, coronavirus disease 2019.

Model 1 was unadjusted. Model 2 was adjusted for age (year, continuous), sex (male or female), and duration between the last vaccination and baseline survey (days, continuous). Model 3 was additionally adjusted for occupation (doctors, nurses, allied healthcare professionals, administrative staff, researchers, and others), risk of occupational exposure to COVID-19 (low, middle, or high), cigarette smoking (never smoker, former smoker, occasional smoker, or current smoker), BMI (kg/m^2, continuous), alcohol drinking (nondrinker, occasional drinker, <1 *go*/day, or ≥1 *go*/day), infection prevention score (continuous), use of public transportation (no or yes), frequency of spending ≥30 minutes without musk in the 3Cs (no, 1 to 2 times, 3 to 5 times, 6 to 9 times, or ≥10 times), frequency of having dinner with ≥5 people for ≥1 hour (no, 1 to 2 times, 3 to 5 times, 6 to 9 times, or ≥10 times), black tea consumption (<1 cup/day, 1 to 2 cups/day, or ≥3

cups/day), number of household (continuous), balanced meal consumption (rarely, 2 to 3 days/week, 4 to 5 days/week, or almost every day), and co-morbid conditions (yes or no). Model 4 was additionally adjusted for SARS-CoV-2 spike antibody titer (AU/ml, continuous).

[a]For coffee consumption model was additionally adjusted for green tea consumption (<1 cup/day, 1 to 2 cups/day, or ≥3 cups/day), and for green tea consumption model was additionally adjusted for coffee consumption (<1 cup/day, 1 cup/day, 2 cups/day, or ≥3 cups/day).

[b]Based on Cox regression analysis, assigning ordinal numbers to the coffee and green tea consumption.
